# Characteristics of Volcanic Tuff from Macicasu (Romania) and Its Capacity to Remove Ammonia from Contaminated Air

**DOI:** 10.3390/molecules27113503

**Published:** 2022-05-30

**Authors:** Marin Senila, Emilia Neag, Oana Cadar, Maria-Alexandra Hoaghia, Marius Roman, Ana Moldovan, Alexandru Hosu, Angela Lupas, Emoke Dalma Kovacs

**Affiliations:** 1 INCDO-INOE 2000, Research Institute for Analytical Instrumentation, 67 Donath Street, 400293 Cluj-Napoca, Romania; emilia.neag@icia.ro (E.N.); oana.cadar@icia.ro (O.C.); alexandra.hoaghia@icia.ro (M.-A.H.); marius.roman@icia.ro (M.R.); ana.moldovan@icia.ro (A.M.); dalma.kovacs@icia.ro (E.D.K.); 2 GeoPlus Services SRL, 99D Braniste Street, 407310 Gilau, Romania; alexandru@geoservplus.com; 3 Doralex Com SRL, 151A Maramureșului Street, 400268 Cluj-Napoca, Romania; office@doralexcom.eu

**Keywords:** zeolite characterization, clinoptilolite, porous materials, ammonia adsorption, air pollution, instrumental analysis

## Abstract

In the present work, the capability of the volcanic tuff from Macicasu (Romania) to remove ammonia (NH_3_) from air with different contamination levels during 24 h of adsorption experiments was investigated. The natural zeolitic volcanic tuff was characterized using X-ray diffraction (XRD), scanning electron microscopy (SEM), the Brunauer–Emmett–Teller (BET) method, inductively coupled plasma optical emission spectrometry (ICP-OES), and thermogravimetric analysis (TGA). The adsorption capacities varied between 0.022 mg NH_3_ g^−1^ zeolite and 0.282 mg NH_3_ g^−1^ zeolite, depending on the NH_3_ concentrations in the air and at the contact time. The nonlinear forms of the Langmuir and Freundlich isotherm models were used to fit the experimental data. Additionally, the adsorption of NH_3_ was studied using nonlinear pseudo-first-order (PFO), pseudo-second-order (PSO), and Elovich kinetic model. Based on the total volume of pores of used volcanic tuff, the NH_3_ was removed from the air both due to the physical adsorption of NH_3_ gas and the ion exchange of NH_4_^+^ (resulted from a reaction between NH_3_ and H_2_O adsorbed by the zeolite). Depending on the initial NH_3_ concentration and the amount of volcanic tuff, the NH_3_ concentrations can be reduced below the threshold of this contaminant in the air. The adsorption capacity of NH_3_ per unit of zeolite (1 g) varied in the range of 0.022–0.282 mg NH_3_ g^−1^ depending on the NH_3_ concentration in the air.

## 1. Introduction

The gas ammonia (NH_3_) can be released in the environment from different sources, but it is estimated that over 90% of NH_3_ emissions in Europe originate from agriculture [1]. Among agriculture sources, one of the most important is represented by the animal husbandry industry, during the natural degradation processes in slurry and manure [2]. Additionally, the application of urea-based fertilizer produced by Haber–Bosch process is an important source of NH_3_ emissions [1].

NH_3_ odor is sharp, intensely irritating, and the threshold for its concentration in air is 18–38 mg m^−3^ (25–53 ppm) [3]. Next to the unpleasant odor, human exposure to excessive NH_3_ gas can cause chemical burns of the respiratory tract, skin, and eyes. In reaction with water, NH_3_ becomes ammonium hydroxide that causes necrosis of the tissues [3]. Furthermore, the released NH_3_ reacts with the nitrogen and sulfur oxides (NO_x_ and SO_x_) existing in air to form particulate matter with a diameter ≤ 2.5 μm (PM2.5) [3]. Studies showed that exposure to NH_3_ gas affect human health by causing respiratory diseases [4].

Thus, owing to the critical risks associated with NH_3_ in air, legislative guidelines were elaborated to set measures for reducing and controlling the NH_3_ emissions from agriculture [5,6]. According to the guidance document on preventing and abating ammonia emissions from agricultural sources [6], nitrogen management should take into consideration the increasing N use efficiency in order to reduce the NH_3_ emissions.

Dropping the N loss leads to increasing the fertilizer value, leading to higher efficiency [7]. Among the possible techniques to reduce the NH_3_ emissions from animal housing are the addition of different additives in slurry and manure. Dropping the pH below a level of 6, with mineral strong acids to reduce NH_3_ emissions, is a practice in some farms [8,9]. However, this practice is hazardous due to the necessity of storage and handling of strong acids that are highly corrosive and may lead to the formation of other toxic compounds. Another possibility is the use of organic acids as amendments, not so much corrosives as mineral acids. Their disadvantages are given by rapid degradation (releasing CO_2_) and by the large amounts necessary to decrease the pH up to the wanted level, because they are typically weak acids [10].

An environmentally friendly alternative to reduce the NH_3_ emissions is the use of adsorbent materials. However, to the best of our knowledge, the information on the efficiency of these materials for NH_3_ gas adsorption capacity is very scarce. Among adsorbent materials, zeolites are natural aluminosilicate materials with porous crystalline structures and well-defined channels or cavities [11,12,13]. They have a three-dimensional network composed of SiO_4_ and AlO_4_ tetrahedra joined by oxygen atoms [14]. Dependent on zeolite type, the Si/Al ratio in its framework is variable, but the Si content is always higher than the Al content [15].

Clinoptilolite is one of the most widespread minerals in the natural volcanic tuffs, suitable for adsorption due to its physicochemical characteristics. Even if is classified in the heulandite group, clinoptilolite has a higher Si/Al ratio (4 ≤ Si/Al < 5.2) than heulandite (Si/Al < 4) and has a better thermal stability (750–800 °C) compared with heulandite (450–550 °C) [16]. Its microporous structure and negative charge provide its ability to adsorb molecules of appropriate cross-sectional diameters, to exchange cations, or to lose and gain water reversibly. Thus, clinoptilolite has molecular sieve capacity, ion-exchange capacity, and catalytic activity [17].

Due to its unique properties, natural zeolites were used in many applications such as soil and water treatment, catalysis, cosmetics, medicine, agronomy, or for gas purification [18,19,20,21,22]. The use of zeolite clinoptilolite as NH_3_ gas removal for prevention of its release from the animal husbandry industry is of great interest. In the majority of existing studies on zeolites, these were used for the removal of ammonium (NH_4_^+^) ions from aqueous solutions, absorbed into the zeolite by an ion-exchange mechanism [23]. Zeolites can adsorb gaseous molecules with a smaller diameter than their internal pores. The adsorption of gaseous NH_3_ onto zeolite is different from NH_4_^+^ because NH_3_ is a neutral molecule, and thus is physically bonded [24]. Some authors reported the development of porous NH_3_ adsorbents with organic parts [25]. However, natural zeolites are inexpensive, widespread materials that are more appropriate for large-scale utilization, and considering the similarities of the chemical behavior of NH_3_ and H_2_O molecules and the good adsorption capacity of water by zeolites, the suitability of zeolite clinoptilolite as NH_3_ adsorbent can be supposed.

This work investigates the removal of NH_3_ from contaminated air using the zeolitic volcanic tuff from Macicasu (Romania), with the main aim of defining the zeolite adsorption capacity. To the best of our knowledge, this is the first work examining the use of this material for NH_3_ adsorption from air. The experiments were carried out in two sealed glass boxes by measuring the NH_3_ concentration in the air sampled from the boxes, with and without zeolite, after NH_3_ generation by an ammonium hydroxide (NH_4_OH) solution, at three different concentration levels.

## 2. Results and Discussion

### 2.1. Volcanic Tuff Characteristics

The zeolitic volcanic tuff was sampled from Macicasu quarry, located in the north-western part of the Transylvanian Depression, Romania [26]. The chemical composition (wt.%) and loss of ignition (LOI) of a whole rock zeolitic tuff sample is presented in Table 1. The measured Si/Al ratio > 4 in the zeolitic tuff sample suggests the clinoptilolite is a major constituent [16].

**Figure 1:**
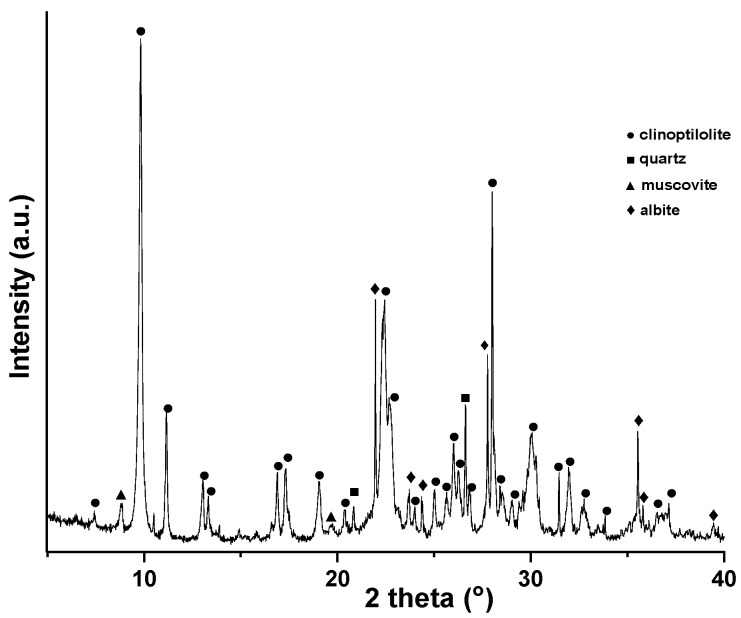
X-ray diffraction patterns of the zeolitic tuff sample.

X-ray diffraction (XRD) analysis shown in Figure 1 indicates the presence of the clinoptilolite as the main zeolite mineral phase of the zeolitic tuff sample. The sample also contains quartz, muscovite, and albite as minor phases.

Since the formation of minerals is determined mainly by the geological, physical, and chemical conditions, the zeolite deposits generally represent a mixture of zeolite minerals and various gangue minerals including quartz, feldspathoids, feldspars, and phyllosilicates (micas, clay minerals) [27]. Accordingly, the XRD pattern of the zeolite sample indicates the presence of clinoptilolite (PDF 00–0147-1870) as the main phase, convoyed by muscovite (PDF 01–073-9867), albite (PDF 01–071-1150), and quartz (PDF 00–046-1045). The XRD pattern of the zeolites exhibits the representative diffraction peaks of the clinoptilolite zeolite structure (2θ around 10, 25, 26, 30, and 32°) [28]. The RIR (Reference Intensity Ratio) method [29] used for the quantitative phase analysis indicates that the zeolite sample contains zeolites (75% clinoptilolite) attended by plagioclase feldspars (8%), silica polymorphs (5%), and clay minerals (11%). The degree of crystallinity of the studied zeolite sample is 84%. The noncrystalline components were not quantified by the XRD analysis, but the presence of amorphous volcanic glass in the zeolite sample is shown by the broad diffraction hump centered at 2θ = 25°. The low amorphous content can be ascribed to the presence of quartz and kaolinized volcanic ash [30].

**Figure 2:**
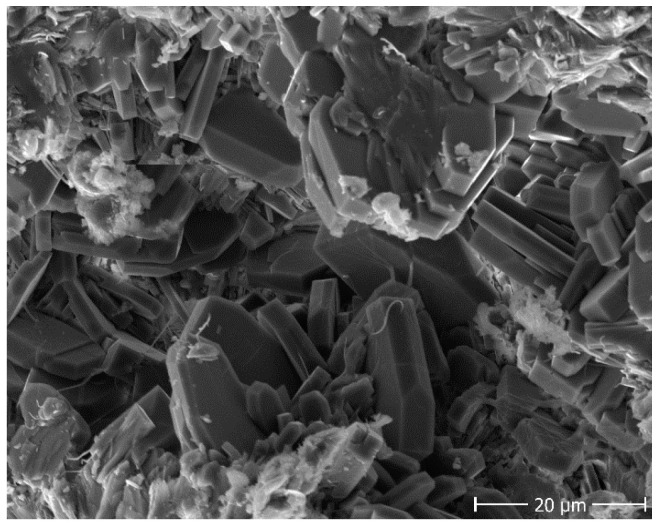
SEM image of the zeolitic tuff sample.

Characterization studies further continued with the SEM–EDX analysis. The image obtained by SEM is shown in Figure 2. As seen, the surface is heterogeneous and, additionally, contains obvious porous structures. Clinoptilolite (tabular crystals) is observed as the main mineral in the sample.

**Figure 3:**
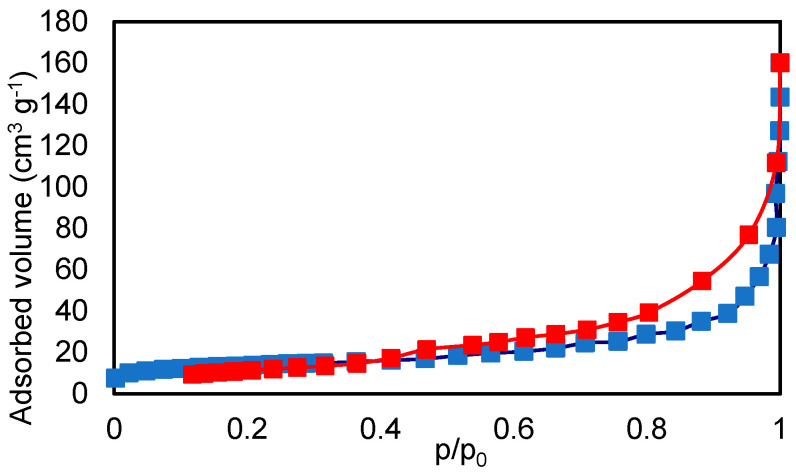
Adsorption-desorption isotherm of the zeolite sample using the BET method. Adsorption data: blue color. Desorption data: red color.

The adsorption–desorption isotherm of the zeolite sample using the BET method is presented in Figure 3. The total surface area measured using the BET method is 46.1 m^2^ g^−1^, the total pore volume is 0.073 cm^3^ g^−1^, and the average pore radius is 36 Å. According to the classification of pores established by the International Union of Pure and Applied Chemistry (IUPAC), all zeolite samples have mesoporous structures due to pore widths less than 50 nm [31].

**Figure 4:**
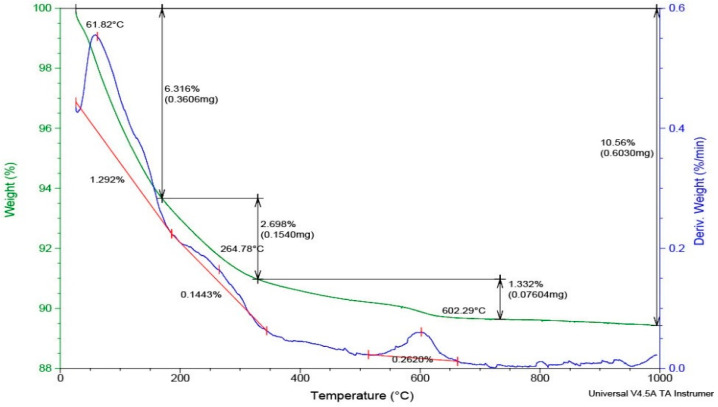
Thermogravimetric analysis (DSC-TGA) of the zeolitic tuff sample.

The thermogravimetric analysis (TGA) of the zeolitic tuff sample presented in Figure 4 was investigated up to 1000 °C. The TGA data indicate a relatively low total weight loss of 10.56%. A 6.31% weight loss was observed up to 150 °C, caused by the gases and water desorption from the sample surface and the beginning of the dealumination processes [32]. Additionally, 2.698% of the total weight is lost at temperatures in the range of 150–350 °C due to the loss of other sample components that are decomposed at low temperatures and the loss of water molecules from the zeolite structure, while 1.332% from the total weight is lost at temperatures of about 600 °C, in the last dehydration stage [31]. The high thermal stability is specific to the clinoptilolite mineral [33].

### 2.2. Experiments for NH_3_ Adsorption of from the Air

#### 2.2.1. Adsorption of NH_3_ Released from 0.5 mL NH_4_OH 25%

**Figure 5:**
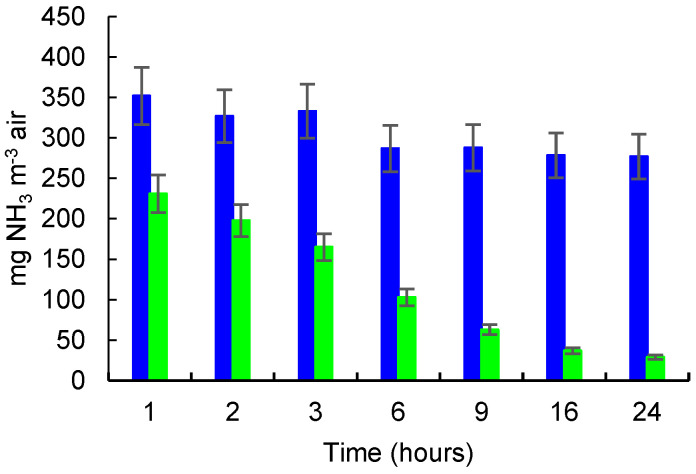
NH_3_ released/adsorbed from 0.5 mL NH_4_OH solution 25%. NH_3_ concentration (mg m^−3^) in control box (blue color) and in box with zeolite (green color); error bars for 10% Ue rel are shown.

**Figure 6:**
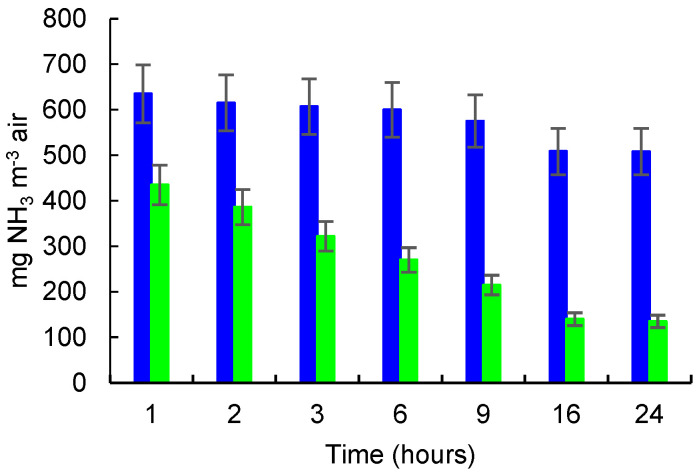
NH_3_ released/adsorbed from 1 mL NH_4_OH solution 25%. NH_3_ concentration (mg m^−3^) in control box (blue color) and in box with zeolite (green color); error bars for 10% Ue rel are shown.

**Figure 7:**
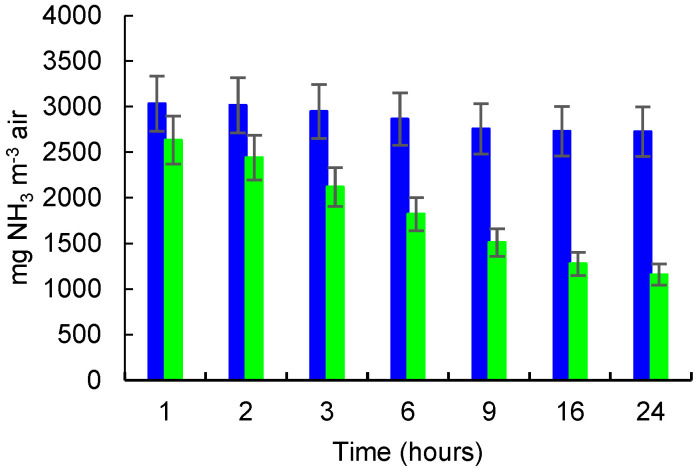
NH_3_ released/adsorbed from 5 mL NH_4_OH solution 25%. NH_3_ concentration (mg m^−3^) in control box (blue color) and in box with zeolite (green color); error bars for 10% Ue rel are shown.

Because clinoptilolite is known to have a good adsorption capacity for H_2_O, and considering the similarity between NH_3_ and H_2_O molecules (and even higher dipolar moment of NH_3_), a good affinity of zeolite toward NH_3_ is also expected to be observed. In this experiment, 0.5 mL of NH_4_OH solution 25% were added into a watch glass and placed into a sealed glass box (volume of 54 L). The concentrations of NH_3_ (mg m^−3^) in the control glass box (A) and the glass box with zeolite (B) determined at 1, 2, 3, 6, 9, 16. and 24 h, respectively, are presented in Figure 5. Three parallel experiments were carried out for each level of NH_3_ concentration and the results represent the average value ± the estimated measurement expanded uncertainty (*Ue*). *Ue* was calculated for a *p* = 95% confidence level by multiplying the cover factor (k = 2) with composed uncertainty (*Uc*). *Uc* was assessed by combining individual uncertainty sources (repeatability of NH_3_ releasing into the box, air sampling, and spectrophotometric NH_3_ determination, evaluated from repeated determination) in the traceability chain. Relative *Ue* for the whole measurement process was evaluated to be 10% from the average measured value. This was included as percent error bars in Figure 5, Figure 6 and Figure 7.

The results shown in Figure 5 indicate that the NH_3_ concentration continuously decreased over time in the glass box containing zeolite from 231 mg NH_3_ m^−3^ after 1 h of incubation to 29 mg NH_3_ m^−3^ after 24 h. According to the presented data, and based on the measurement uncertainty, the differences between NH_3_ concentrations in the control box and those measured in the control box at each measurement time are statistically significant. In the control box, the concentration of NH_3_ also decreased over time, but with a much smaller rate, from 352 mg NH_3_ m^−3^ after 1 h of incubation to 277 mg NH_3_ m^−3^ after 24 h. In total, a decrease of approximately 21% of the NH_3_ concentration in the control box was observed from 1 h to 24 h, which is probably caused by the decreasing of the NH_3_ amount due to the sampling, and possible ammonia precipitation processes during this period. If the measurement uncertainty is accounted for by the average NH_3_ concentrations in the control box, the differences between the successive measurements in time are not statistically significant. However, the measured NH_3_ concentration at 1 h is different from that measured at 24 h, which confirms the general decreasing tendency.

#### 2.2.2. Adsorption of NH_3_ Released from 1 mL NH_4_OH 25%

The NH_3_ released from 1 mL of NH_4_OH solution 25% in the control glass box and the NH_3_ concentrations in the glass box with zeolite (B) measured during the incubation time of 24 h are presented in Figure 6.

In this experiment, the NH_3_ concentration decreased over time in the glass box containing zeolite from 435 mg NH_3_ m^−3^ after 1 h of incubation to 135 mg NH_3_ m^−3^ after 24 h, while in the control box, the concentration of NH_3_ decreased from 635 mg NH_3_ m^−3^ after 1 h of incubation to 508 mg NH_3_ m^−3^ after 24 h of incubation. A reduction with approximately 20% of the NH_3_ concentration in the control box was observed from 1 h to 24 h of incubation. The differences between NH_3_ concentrations in the control box and those measured in the control box at each measurement time are statistically significant if the expanded uncertainty of 10% are considered for the average values.

#### 2.2.3. Adsorption of NH_3_ Released from 5 mL NH_4_OH 25%

The NH_3_ released from 5 mL of NH_4_OH solution 25% in the control glass box and the NH_3_ concentrations in the glass box with zeolite (B) measured during the incubation time of 24 h are presented in Figure 7.

When 5 mL NH_4_OH 25% were introduced in experimental boxes, the NH_3_ concentration was 3034 mg NH_3_ m^−3^ after 1 h of incubation, and continuously decreased over time to 2726 mg NH_3_ m^−3^ after 24 h of incubation in the control box. In the glass box containing zeolite, the concentration was 2634 mg NH_3_ m^−3^ after 1 h and decreased to 1158 mg NH_3_ m^−3^ after 24 h. The differences between NH_3_ concentrations in the control box and those measured in the control box at each measurement time are, in general, statistically significant, for an expanded uncertainty of 10%. However, for the first measurement period (1 h), the difference between the NH_3_ concentrations in the control box and in the box with zeolite is not yet significant.

#### 2.2.4. Sorption Rate (Rs%) of NH_3_ Released from 0.5 mL, 1 mL, and 5 mL NH_4_OH 25%

The sorption rates (Rs%) of the NH_3_ eliminated from the air contaminated with 0.5 mL, 1 mL, and 5 mL NH_4_OH 25%, calculated for each sampling period using the Equation (1), are presented in Table 2.

The sorption rate (Rs%) increased from 34.4% after 1 h of experiment to 89.5% after 24 h for the NH_3_ adsorption from air contaminated with 0.5 mL NH_4_OH 25%. For the experiment with air contaminated with 1 mL NH_4_OH 25%, Rs% increased from 31.5% after 1 h of experiment to 73.4% after 24 h while in the case of air contaminated with 5 mL NH_4_OH 25%, Rs% was already 13.2% after 1 h of experiment and increased to 57.5% after 24 h.

#### 2.2.5. Adsorption Capacity

**Figure 8:**
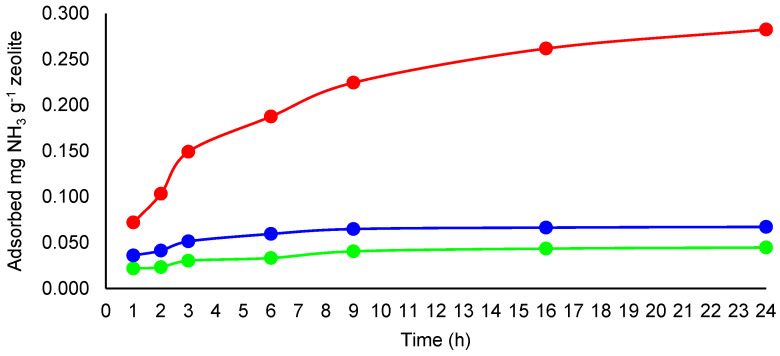
Adsorption capacity (mg NH_3_ g^−1^ zeolite) for the NH_3_ adsorbed during the 24 h period of experiments from air contaminated with 0.5 mL NH_4_OH 25% (green line), 1 mL NH_4_OH 25% (blue line), and 5 mL NH_4_OH 25% (red line).

The adsorption capacity for NH_3_ per unit of zeolite (1 g) was calculated using Equation (2), considering the difference between the concentration of the NH_3_ released in the control glass box and the concentration of the NH_3_ measured in the glass box containing zeolite (*C_A-B_*), the volume of the testing boxes, and the amount of the zeolite introduced into the box for adsorption. The evolution of the adsorbed NH_3_ during the 24 h of experiments is presented in Figure 8.

In the experiment with 0.5 mL NH_4_OH 25% added in the boxes, *A_C_* increased from 0.022 mg g^−1^ after 1 h of incubation time to 0.045 mg g^−1^ after 24 h of the adsorption experiment. When 1 mL NH_4_OH 25% was introduced in the boxes, *A_C_* increased from 0.036 mg g^−1^ after 1 h to a maximum of 0.065 mg g^−1^ after 9 h of the adsorption experiment, then this value remained almost unchanged until the end of the experiment. In the experiment with 5 mL NH_4_OH 25% introduced in the boxes, *A_C_* increased at 0.072 mg g^−1^ after 1 h of incubation time and increased continuously to 0.282 mg g^−1^ after 24 h of adsorption time. These results indicated that with the growing of NH_3_ concentration in the air, the adsorbed amount of NH_3_ also increases, even if the sorption rate (%) is smaller.

The calculated values for NH_3_ removed from air per g of zeolite were in the similar order of magnitude with those reported for adsorption capacity of clinoptilolite between 0.09 mg g^−1^ and 0.13 mg g^−1^ [24], but were smaller than the ammonia adsorption capacity up to the saturation point for Iranian natural clinoptilolite, in the range of 6.255–14.155 mg N g^−1^ reported by Asilian et al. [34]. However, the results reported by Asilian et al. [34] were obtained using a different experimental set-up, with zeolite being used in bed columns, in dynamic NH_3_ sorption experiments. In a study on synthetic zeolite, Lucero et al. [35] reported a NH_3_ loading of 1.66–11.71 mmol g^−1^. In our case, the experimental set-up was built to assure the interaction of contaminated air with zeolite only by the natural convection of air to simulate the case of NH_3_ adsorption by zeolite from animal housing.

Considering the total pore volume of the zeolitic tuff sample used in our study of 0.073 cm^3^ g^−1^ (in standard conditions of ideal gas), and assuming that all the pore volume is occupied by NH_3_ molecules, the maximum amount of NH_3_ that can be adsorbed per g of zeolite in our experimental conditions (22 °C and 1.001 atm) was calculated to be 0.0030 mmol (0.0513 mg NH_3_). In the first experiment (0.5 mL NH_4_OH solution 25% introduced into the box), the amounts of removed NH_3_ per g of zeolite were in the range of 0.022–0.045 mg g^−1^ (below 0.0513 mg g^−1^). However, in the case of the experiments with higher amounts of NH_3_ in the air, the amounts of removed NH_3_ per g of zeolite overreached this value (0.0513 mg g^−1^). Thus, it can be observed that the removal of NH_3_ from air is not only due to its adsorption as a gas. Zeolite also adsorbs water molecules from the air (the experiments were conducted in a relative humidity of 64 ± 2%); thus, part of NH_3_ is transformed into NH_4_^+^ according to the reaction from Equation (1):

NH_3_ + H_2_O « NH_4_^+^ + OH^−^ (1)

Accordingly, the role of clinoptilolite is not only to adsorb NH_3_ molecules by physical adsorption (related with the framework structure of clinoptilolite, caused by external molecular force and electrostatic force, characteristic of the common adsorption path of porous materials) [24], but also NH_4_^+^, which is adsorbed by cation exchange processes [36]. In a previous study on the zeolitic volcanic tuff from the Macicas deposit, Maicaneanu and Bedelean reported cation adsorption capacities between 5.42 and 33.8 mg NH_4_^+^ g^−1^, obtained by experiments in a fixed bed column; thus, this material has a high cation exchange capacity for NH_4_^+^ [37].

### 2.3. Isotherm Modeling

**Figure 9:**
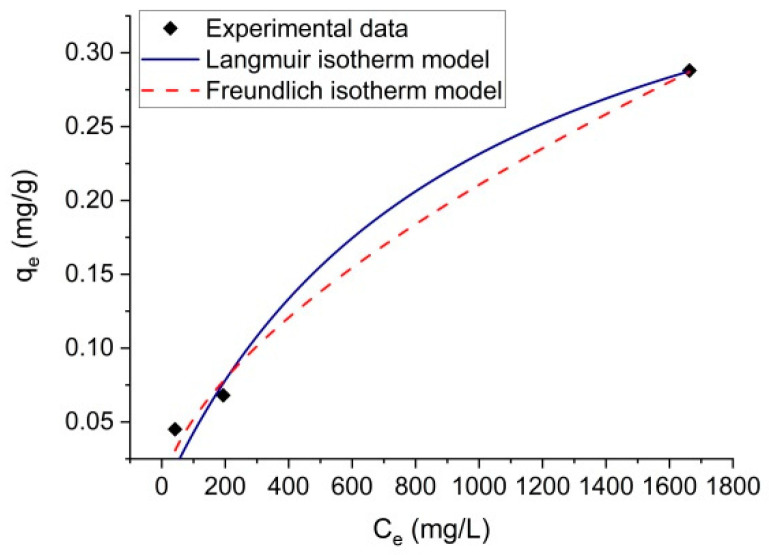
Plots of the Langmuir and Freundlich isotherm models for NH_3_ adsorption onto thermally treated volcanic tuff.

The plots of the Langmuir and Freundlich isotherm models for NH_3_ adsorption onto thermally treated volcanic tuff are presented in Figure 9.

The Langmuir and Freundlich isotherm constants and correlation coefficients (R^2^) are presented in Table 3. The highest value of R^2^ was obtained when the experimental data were fitted into the Freundlich isotherm model (R^2^ = 0.9916), as compared with the Langmuir isotherm model (R^2^ = 0.9793). The value of *q_max_* determined using the Langmuir isotherm model was 0.45, higher than the experimental *q_e_* values (*q_e, exp_)*. Thus, the Langmuir isotherm model is not suitable to describe the experimental data for NH_3_ adsorption onto thermally treated volcanic tuff. The results suggested that the adsorption mechanism of NH_3_ followed the Freundlich isotherm model, implying that the thermally treated volcanic tuff has a heterogeneous surface.

### 2.4. Kinetic Modeling

The PFO, PSO, and Elovich constants and their corresponding R^2^ values are listed in Table 4. The obtained values of R^2^ indicated that the nonlinear form of PFO fitted the experimental results. The nonlinear form of PSO exhibited slightly low R^2^ (0.9620, 0.9485, and 0.9868) for the adsorption of NH_3_ onto thermally treated volcanic tuff, as compared with the nonlinear form of PFO (0.9653, 0.9772, and 0.9871). Additionally, the results suggested that the calculated *q_e_* values (*q_e, calc_)* obtained using the nonlinear form of PFO model were closer to the experimental *q_e_* values *q_e (exp)_*. Thus, the nonlinear form of the PFO model is more adequate for describing the kinetics adsorption of NH_3_ onto thermally treated volcanic tuff than PSO model.

Table 4 also showed that the R^2^ values for the Elovich kinetic model were slightly lower than those obtained for the PFO kinetics model for NH_3_ eliminated from the air contaminated with 1 mL and 5 mL NH_4_OH 25%.

Gebreegziabher et al. [38] studied the H_2_S, NH_3_, and (CH_3_)_3_N adsorption from indoor air using a porous corncob activated carbon. The findings suggested that the nonlinear form of PSO model showed a satisfactory correlation with a high coefficient of determination of 0.9978 for NH_3_ sorption onto porous corncob activated carbon.

## 3. Materials and Methods

### 3.1. Materials

All used reagents were of analytical grade: 37% HCl, 65% HNO_3_, 40% HF, and 25% NH_4_OH (m/m), purchased from Merck (Darmstadt, Germany). Ultrapure water was obtained from a Milli Q system (Millipore, France) and used for the dilutions. The accuracy of the analysis for total metals concentrations in zeolite was assessed using CRM BCS-CRM 375/1 soda feldspar (Bureau of Analyzed Samples, Middlesbrough, UK). Satisfactory percent recoveries (%) for all the analyzed metals, in the range of 88–102%, were obtained.

Zeolitic volcanic tuff material sampled from a quarry located in Macicasu, Cluj County, Romania, was crushed and sieved to obtain a particle size < 100 µm, and then thermally treated at 200 °C for 3 h.

### 3.2. Characterization

The volcanic tuff was characterized regarding the chemical composition in the whole sample for major elements using ICP–OES after microwave-assisted digestion with a mixture of HNO_3_ 65%:HCl 37%:HF 40% (3:9:2, *v:v:v*). The measured concentrations of major elements (Al, Fe, Na, K, Ca, and Mg) were converted to oxides using atomic and molecular masses. The cation exchange capacity (CEC) was determined after the ammonium acetate saturation (AMAS) extraction method and after measuring the extractable major cations (K, Na, Ca, and Mg) using ICP-OES Optima 5300 DV (Perkin Elmer, Waltham, MA, USA). The operating conditions used for ICP–OES determination were 1300W RF power, 15 L min^−1^ Ar plasma support, 2.0 L min^−1^ auxiliary Ar flow, 0.8 L min^−1^ nebulization Ar, and 1.5 mL min^−1^ sample uptake rate. In addition, 7-point linear calibration curves over the range 0–10 mg L^−1^ element were plotted. Calibration standards were prepared from ICP multielement standard IV solution 1000 mg L^−1^ (Merck, Darmstadt, Germany) by appropriate dilutions. SiO_2_ from volcanic tuff was determined gravimetrically. The X-ray diffraction (XRD) patterns were recorded at room temperature using a D8 Advance (Bruker, Karlsruhe, Germany) diffractometer with CuKα radiation (λ = 1.54060 Å), operating at 40 kV and 40 mA. To evaluate the morphology, the zeolites were analyzed using the scanning electron microscope SEM FEIXL30SFEG upgraded to remX Microscope Control (Thermo Electron Corporation, Waltham, MA, USA) with a microanalytical system (EDS) Oxford Aztec Advanced system (Oxford Instruments, Abingdon, Oxfordshire, UK). Total surface area and pore radius were obtained from N_2_ adsorption–desorption isotherms using the Brunauer–Emmett–Teller (BET) method for total surface area evaluation, and Dollimore–Heal model for porosity data. The isotherms were obtained using a Sorptomatic 1990 apparatus (Thermo Electron Corporation, Waltham, MA, USA).

### 3.3. Experimental Set-Up

Two similar glass boxes with dimensions of 60 × 30 × 30 cm (L × W × D), with a volume of 54 L, were used in the study. The experiments were conducted at room temperature (22 ± 2 °C) in a relative moisture measurement of 64 ± 2%. To evaluate the zeolite adsorption capacity for NH_3_ released in the air, 0.5, 1, and 5 mL of NH_4_OH solution 25% (*m*/*m*) were introduced in a watch glass in each glass box. In one box, an amount of 300 g zeolite (particle size <100 µm) was introduced in a 20-micron Nylon mesh material, suspended from the top part, inside the box. The boxes were hermetically sealed before and after air sampling. At different periods of time, 15 L air samples were taken from each box through a small orifice, using the sampling pump. The air was sampled into an adsorbing solution containing H_2_SO_4_ 0.01 N with a flow-rate of 3 L min^−1^ for 5 min using a sampling pump HSF-513 AUP Gilian (Sensidyne, St. Petersburg, FL, USA). NH_3_ concentration was spectrophotometrically determined at a wavelength of 450 nm after a reaction with Nessler reactive, using a Lambda 25 (Perkin Elmer, Waltham, MA, USA) UV-VIS spectrophotometer.

### 3.4. Sorption Rate (Rs%) and Adsorption Capacity Calculation

The sorption rate (Rs%) of the NH_3_ eliminated from the air was calculated for each sampling period according to the following formula:

*Rs* = *C_A-B_* * 100/*C_A_* (2)

in which *C_A-B_* is the difference between the concentration of the ammonia released in the control glass box and the concentration of the NH_3_ measured in the glass box containing zeolite at the same sampling period, while *C_A_* is the concentration of the NH_3_ measured in the control glass box.

The adsorption capacity for NH_3_ per unit of zeolite (1 g) was calculated using the Equation (3):

*A_C_* = *C_A-B_* * *V*/(1000 * *m*) (3)

in which *A_C_* represents the adsorption capacity for NH_3_ per gram of zeolite, *V* is the volume of glass box (54 L), and *m* is the mass of the adsorbing zeolite (300 g).

### 3.5. Isotherm Modeling

The experimental equilibrium data were fitted using nonlinear forms of the Langmuir and Freundlich isotherm models. The parameters were determined using OriginPro software, version 2020b, OriginLab Corporation, Northampton, MA, USA.

The Langmuir isotherm model in its nonlinear form is expressed as [39,40,41]:

(4) $$q_{e}=\frac{q_{max}K_{L}C_{e}}{1+K_{L}C_{e}}.\;$$

in which *q_max_* is the maximum adsorption capacity (mg g^−1^), *C_e_* is the NH_3_ concentration at equilibrium (mg L^−1^), and *K_L_* is the Langmuir constant (L mg^−1^) [40].

The Freundlich isotherm in its nonlinear form is expressed as [40,41,42]:

(5) $$q_{e}=K_{F}C_{e}{}^{\left(1/n\right)}$$

in which *K_F_* is related to adsorption capacity (mg^1−1/n^ L^1/n^ g^−1^) and *n* is related to adsorption intensity [40].

### 3.6. Kinetic Modeling

In order to describe the kinetics of NH_3_ removal by thermally treated volcanic tuff, the nonlinear forms of pseudo-first-order (PFO) [43], pseudo-second-order (PSO) [44], and the Elovich kinetic model were applied.

The nonlinear form of PFO [41,45] is given as follows:

(6) $$q_{t}=q_{e}\left(1-e^{-k_{1}t}\right)$$

in which *q_e_* is the NH_3_ amount adsorbed at equilibrium (mg g^−1^), *q_t_* is the NH_3_ amount adsorbed at time *t* (mg g^−1^), and *k_1_* is the first order rate constant (min^−1^) [45].

The nonlinear form of PSO [41,45,46] is given as follows:

(7) $$q_{t}=\frac{q_{e}^{2}k_{2}t}{1+q_{e}k_{2}t}$$

in which *k*_2_ is the second order rate constant (g mg·min^−1^) [45].

The nonlinear form of the Elovich kinetic model (chemisorption kinetics), is expressed as [41]:

(8) $$q_{t}=\frac{1}{\beta }\ln\left(\alpha \beta t+1\right)$$

in which *α* is the initial adsorbate rate (mg g min^−1^) and *β* is the desorption constant (g mg^−1^) [41].

The NH_3_ adsorption, *q_e_* was calculated using the following equation:

(9) $$q_{e}=\frac{V_{c}}{m}\cdot \frac{M_{w}}{V_{m}}\cdot (C_{i}-C_{f})$$

in which *V_C_* is the volume of adsorption glass chamber (m^3^), *m* is the mass of zeolite (g), *M_w_* is the molar mass of gas (g mol^−1^), *V_m_* is the molar volume of gas (24 L mol^−1^) at 20 °C, *C_i_* is the initial NH_3_ concentration (mg L^−1^),S and *C_e_* is the equilibrium NH_3_ concentration (mg L^−1^) [38].

## 4. Conclusions

The removal of NH_3_ from controlled, contaminated air using the zeolitic volcanic tuff from Macicasu (Romania) was studied. The experiments were carried out during 24 h of adsorption in two sealed glass boxes by measuring the NH_3_ concentration in air sampled from boxes with and without zeolite, after NH_3_ generation at three different levels of concentrations (0.5 mL, 1 mL, and 5 mL NH_4_OH solution). The sorption rate of air–zeolite after 24 h varied in the following order: 89.5% (0.5 mL NH_4_OH 25%) > 73.4% (1 mL NH_4_OH 25%) > 57.5% (5 mL NH_4_OH 25%). The adsorption capacity of NH_3_ per unit of zeolite (1 g) varied in the range of 0.022—0.045 mg NH_3_ g^−1^ for 0.5 mL NH_4_OH, 0.036—0.067 mg NH_3_ g^−1^, 1 mL NH_4_OH, and 0.072—0.282 mg NH_3_ g^−1^ when 5 mL NH_4_OH was introduced in the box. It was observed that the nonlinear form of the Freundlich isotherm model described the adsorption process. Additionally, the findings revealed that the experimental data followed the nonlinear form of PFO instead of the nonlinear form of PSO. Moreover, the obtained results indicated that NH_4_^+^ resulted from NH_3_ reaction with H_2_O adsorbed by zeolite from air is also retained by ion exchange.

## Figures and Tables

**Table 1:** Chemical composition of zeolitic tuff from Macicasu quarry (n = 3 parallel determinations).

Parameter	Average Value ± stdev.
pH	8.35 ± 0.30
CEC (meq 100 g^−1^)	129 ± 6.5
SiO_2_ (%)	69.14 ± 2.23
Al_2_O_3_ (%)	13.18 ± 0.44
CaO (%)	3.90 ± 0.06
MgO (%)	0.57 ± 0.03
K_2_O (%)	1.02 ± 0.12
Na_2_O (%)	1.10 ± 0.05
Fe_2_O_3_ (%)	1.99 ± 0.06
MnO (%)	0.03 ± 0.004
LOI (%)	9.03 ± 0.90

**Table 2:** Sorption rate for NH_3_ eliminated from air contaminated with 0.5 mL, 1 mL, and 5 mL NH_4_OH 25%.

Measurement Time (h)	Rs% 0.5 mL NH_4_OH	Rs% 1 mL NH_4_OH	Rs% 5 mL NH_4_OH
1	34.4	31.5	13.2
2	39.4	37.2	19.0
3	50.5	47.0	28.1
6	64.1	55.0	36.4
9	78.1	62.6	45.2
16	86.7	72.5	53.2
24	89.5	73.4	57.5

**Table 3:** Langmuir and Freundlich isotherm parameters and correlation coefficients of NH_3_ adsorption onto thermally treated volcanic tuff obtained by nonlinear fitting.

Langmuir Isotherm			Freundlich Isotherm		
q_max_	K_L_	R^2^	K_F_	n	R^2^
(mg g^−1^)	(L mg^−1^)		(mg ^1−1/n^ L^1/n^ g^−1^)		
0.45	0.001	0.9793	0.003	0.61	0.9916

**Table 4:** PFO, PSO, and Elovich parameters and correlation coefficients of NH_3_ adsorption onto thermally treated volcanic tuff obtained by nonlinear fitting.

Model	Parameters	0.5 mL NH_4_OH	1 mL NH_4_OH	5 mL NH_4_OH
PFO	*k*_1_ (1 min^−1^)	0.008	0.01	0.004
	*q_e, calc_* (mg g^−1^)	0.043	0.067	0.275
	R^2^	0.9653	0.9772	0.9871
PSO	*k*_2_ (g mg·min^−1^)	0.225	0.208	0.014
	*q_e, calc_* (mg g^−1^)	0.048	0.073	0.328
	R^2^	0.9620	0.9485	0.9868
Elovich	*α* (mg g min^−1^)	0.002	0.007	0.003
	*β* (g mg^−1^)	123.32	94.99	12.98
	R^2^	0.9872	0.9694	0.9844
	*q_e, exp_* (mg g^−1^)	0.045	0.068	0.288

## Data Availability

Not applicable.

## Sample Availability

Samples are available from the authors.
